# The Long-Term Impact of Therapeutic Fasting on Primary Dysmenorrhea in Young Female Adults: A Randomized Controlled Trial

**DOI:** 10.7759/cureus.41437

**Published:** 2023-07-06

**Authors:** Saraswati Tewani, Hemanshu Sharma, Gulab R Tewani, Prakash B Kodali, Pradeep MK Nair

**Affiliations:** 1 Department of Naturopathy, Sant Hirdaram Medical College of Naturopathy and Yogic Sciences for Women, Bhopal, IND; 2 Department of Community Medicine, Sant Hirdaram Medical College of Naturopathy and Yogic Sciences for Women, Bhopal, IND; 3 Department of Naturopathy, Sant Hirdaram Yoga and Nature Cure Hospital, Bhopal, IND; 4 Department of Public Health and Community Medicine, Central University of Kerala, Kasaragod, IND; 5 Department of Research, Sant Hirdaram Medical College of Naturopathy and Yogic Sciences for Women, Bhopal, IND

**Keywords:** pain, gynecological disorders, nutrition, menstrual pain, menstrual distress, fasting

## Abstract

Background and objective

Primary dysmenorrhea (PD) is one of the leading health issues among women. According to reports, nutrition/diet significantly affects the severity and course of PD. The present study aimed to evaluate the role of therapeutic fasting in alleviating the symptoms associated with PD, improving quality of life (QoL), and reducing absenteeism among young female adults with PD.

Methods

A total of 52 participants aged between 18 and 24 years were included in the study and randomly classified into two groups of 26 each. The study group (fasting group) was assigned to undergo a 10-day fasting regimen (≤500 kcal/day) while the control group was to follow a normal dietary routine. The severity of pain, associated distress symptoms, QoL, and sleep quality were measured at baseline and on the fifth day of the first, second, and third menstruation cycles after the intervention, using the Visual Analog Scale (VAS), Numerical Rating Scale (NRS), the World Health Organization Quality of Life Brief Version (WHOQOL-BREF) questionnaire, and Pittsburg Sleep Quality Index. We also recorded the rate of absenteeism among the study participants during menstruation cycles.

Results

We observed a significant reduction in pain (p<0.001), cramps (p=0.001), nausea/vomiting (p=0.02), dizziness (p=0.007), and mood changes (p=0.005) in the study group compared to the control group. The effects were persistent in most of the variables at the second and third follow-ups as well. The physical (p=0.005) and psychological (p<0.001) QoL significantly improved in the first month, and we observed a similar trend at the second (physical p=0.03; psychological p=0.001) and third follow-ups (psychological p=0.002) except for physical QoL, which was significant only at the second follow-up. The fasting group had significantly lower absenteeism compared to the controls during the first follow-up (p=0.001).

Conclusion

Therapeutic fasting may be considered a safe and effective option in the management of PD. Future trials should examine the long-term sustainability of the results.

## Introduction

Primary dysmenorrhea (PD) is an underdiagnosed and poorly managed condition among young female adults. Moreover, it is significantly associated with poor quality of life (QoL), absenteeism, and physical and mental distress [1]. The prevalence of dysmenorrhea among Indian adolescents and women ranges from 50 to 87%, indicating that PD is a very common issue encountered by Indian women [2]. Conventionally, PD is managed with non-steroidal anti-inflammatory drugs (NSAIDs) and hormonal contraceptives [1]. Alternatively, reports have revealed the use of non-pharmacological interventions like exercise, dietary modifications, aromatherapy, acupuncture, and thermal therapies among PD patients [3].

A recent systematic review has indicated the positive role of nutrition and diet in alleviating menstrual pain. However, this review has also highlighted the need for conducting further interventional studies due to the methodological limitations in the existing literature [4]. Fasting is one such modified diet program that is becoming increasingly popular among researchers and healthcare providers. Because of its multidimensional impact on various disorders among women, studies suggest that therapeutic fasting (medically supervised fasting) is beneficial in managing these disorders [5,6].

Many studies have found that therapeutic fasting increases serotonin levels, endogenous opioids, and endocannabinoids [7,8]. Furthermore, therapeutic fasting is associated with enhanced mood [7] and an improvement in QoL [9]. These properties of therapeutic fasting in alleviating pain, improving mood, and enhancing QoL can play a significant role in the management of PD. However, to date, no studies have been conducted to substantiate the use of therapeutic fasting in PD.

In light of this, the present study hypothesized that a 10-day therapeutic fasting regimen would be beneficial in reducing the symptoms associated with PD and improving the QoL among young female adults with PD.

## Materials and methods

Study setting and ethical considerations

The study was conducted at a private medical college in Bhopal, India, and the participants were medical students from the same college. This project was approved by the Institutional Ethics Committee of the institution and registered in the Clinical Trial Registry of India. All the participants signed a written informed consent form before the commencement of the study.

Study design

This was an open-label, pilot randomized controlled trial with two arms: one that underwent a 10-day regimen of supervised therapeutic fasting and one that followed a regular routine with menstrual hygiene advice.

Study participants

The participants were undergraduate students who were pursuing their medical degrees at the study institution and were diagnosed with PD based on a thorough case history. All the participants underwent a screening through ultrasonography to eliminate any pelvic pathology. They were screened for information regarding age, date of menarche, duration of menstrual cycle, and duration of menstrual flow and classified on the basis of severity of dysmenorrhea: mild dysmenorrhea (1-3 points), moderate dysmenorrhea (4-7 points), and severe dysmenorrhea (8-10 points) by Visual Analog Scale (VAS) and confirmed by detailed clinical history. The inclusion and exclusion criteria for the study were as follows:

Inclusion Criteria

The study included females between the ages of 18 and 24 years who had PD for at least three months and were classified as having moderate dysmenorrhea (4-7 points) or severe dysmenorrhea (8-10 points) and screened by VAS for pain and having a menstrual period of three to seven days.

Exclusion Criteria

We excluded participants if they (a) had a contributing diagnosis for endometriosis and adenomyosis diagnosed through ultrasonography or were known cases of secondary dysmenorrhea; (b) were suffering from systemic illness and other comorbidities; (c) were using intrauterine contraceptive devices or oral contraceptive pills; (d) had any medical conditions that prevented them from undertaking fasting; (e) were using conventional or alternative treatments for pain reduction; or (f) were undergoing hormonal replacement therapy.

Sample size

The total sample size was calculated to be n=52 by assuming an effect size of d=0.8, power (1-β) of 80%, and level of significance (α) of 0.05 by using G Power Software version 3.1.9.7.

Randomization

**Figure 1:**
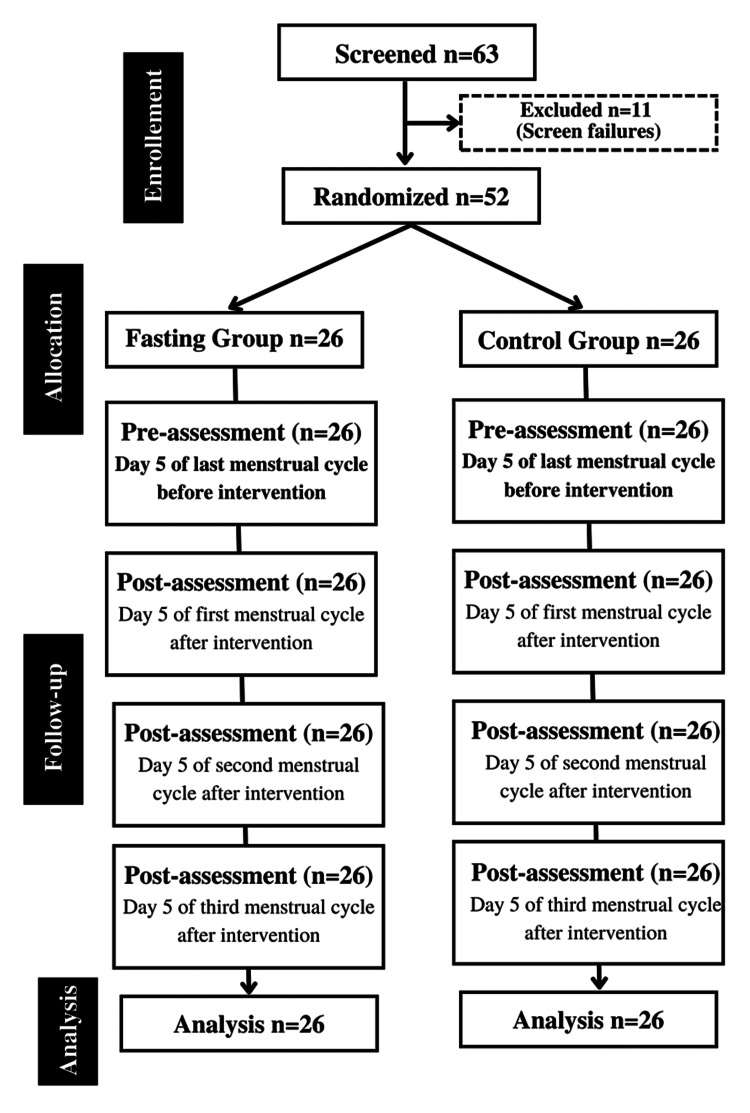
Trial profile

After the selection, participants were randomized, by using the random numbers generated from the computer randomization software, into either an experimental or control group at a ratio of 1:1. The allocation was concealed by using an opaque envelope, and the statistician was blinded to the allocation. The sealed envelope was opened by one of the investigators after the patients signed the informed consent form and then they were assigned to the respective group. The detailed profile of the trial is outlined in Figure 1.

Intervention

Experimental Arm

The experimental arm undertook a 10-day therapeutic fasting program following the Buchinger style, during which participants consumed 2.5 liters of water, vegetable broth, fruit or vegetable juices, and honey, providing approximately 250-500 kcal/day. Additionally, simple therapies like enema and abdominal packs were used to promote elimination through the intestines, liver, kidneys, lungs, and skin [10]. The fasting regimen consisted of three phases: (i) the preparatory phase (1.5 days), where the participants consumed a vegetarian diet (approximately 800-1000 kcal/day); (ii) the fasting phase (seven days); and (iii) the refeeding phase (1.5 days), where the participants returned to their normal routine diet (approximately 800-1000 kcal/day).

Prior to fasting, the experimental group was educated and mentally and emotionally prepared regarding the procedure, rules, and regulations to be followed during the fast. They were counseled by the investigators on the common mild adverse reactions or healing crises that can occur during the fast. They were advised to abstain from consuming any stimulants like caffeine, tea, nicotine, and alcohol. Furthermore, they were advised to perform mild-to-normal physical activities like walking and yoga (meditation, breathing exercises, or relaxing postures). The experimental group's participants received an enema for the first three days of fasting to improve peristaltic movements, as recommended by the international consensus on fasting therapy [10].

Simple naturopathic remedies like cold water sponging, cold compresses, steam inhalation, salt water gargling, warm water drinking, hot foot immersion, acupressure, leg packs, and mud packs were also provided to those participants in the experimental group who reported symptoms like fever, cold, cough, body ache, headache, nausea, and vomiting during the fasting period. Furthermore, the experimental group participants were provided with follow-up measures regarding dos and don’ts during the follow-up period (see Appendices).

Control Arm

The participants in the control arm followed their normal routine during the intervention phase. They were provided with health-related counseling on maintaining menstrual hygiene and lifestyle practices like good sleep, diet, and exercise.

Outcome measures

Outcome measures were evaluated for pain, associated distress symptoms, QoL, absenteeism, and sleep quality. The assessments were carried out at baseline (the fifth day of the last menstruation cycle before the initiation of intervention) and post-intervention (the fifth day of the first menstruation cycle post-intervention), followed by a second and third follow-up on the fifth day of the second and third menstruation cycles post-intervention, respectively. The following tools were used to assess the primary and secondary outcome measures:

Primary Outcome Measure

VAS for pain: the VAS is a 10-point numerical pain rating scale, designed for the purpose of rating pain intensity by drawing a horizontal 10 cm line in the center of a white sheet with "0" representing no pain and "10" signifying the worst possible pain [11]. The participants were asked to indicate the pain intensity by placing a dot on the line to measure pain intensity. 

Secondary Outcome Measures

Numerical Rating Scale (NRS): a 4-point NRS was used for assessing cramps and systemic symptoms like diarrhea, nausea, vomiting, mood changes, and dizziness [12]. This scale has a score range of 0-3, with "0" indicating no symptoms, "1" indicating mild symptoms, "2" indicating moderate symptoms, and "3" indicating severe symptoms.

Pittsburg Sleep Quality Index (PSQI): PSQI is a psychometrically validated, self-reported, and effective instrument used to assess the quality and patterns of sleep. A PSQI score of "5" or more indicates "poor" sleep [13].

The World Health Organization Quality of Life Brief Version(WHOQOL-BREF)*: *WHOQOL-BREF is a 26-item instrument with four domains: physical health (seven items), psychological health (six items), social relationships (three items), and environmental health (eight items), which are scored on a response scale ranging from 1 to 5, after which the scores are linearly summed up to give a final score between 0 and 100 [14].

Absenteeism

College absenteeism was tracked using an attendance sheet, on which participants mark their attendance during the menstruation period.

Data analysis

The analysis was conducted to determine the effectiveness of 10-day medically supervised fasting in reducing the symptoms of PD and improving QoL. The data were refined prior to the analysis and checked for inconsistencies and missing elements. No missing data or data entry errors were found. The fitness of the study data for the assumptions of the statistical test was ensured prior to the analysis. The study characteristics at baseline between the intervention and control groups were compared using two-sample Kolmogorov-Smirnov tests to identify any potential differences between the groups at baseline. The normality of the variables at baseline and follow-up was assessed using the Shapiro-Wilk test [15]. Levene's test was used to assess the homogeneity of variance between the intervention and control groups.

For the normally distributed variables, we used multivariate analysis of covariance (ANCOVA) to study the main effect of the intervention (i.e., the 10-day medically supervised fasting) on the key study variables. The intervention was the independent variable, and pain, QoL, sleep quality, symptoms (cramps, nausea, vomiting, etc.), and absenteeism were the dependent variables. The respective baseline measures of the dependent variables were adjusted in each ANCOVA model. For the variables that were not normally distributed, we used the Mann-Whitney U test to assess if there was a significant difference in the study variables across the intervention and control groups. To identify the long-term effect of the intervention, we analyzed the findings at the second and third follow-ups using the same procedures. We computed effect sizes employing Cohen’s d using the formula, where the means of the intervention and control groups were the pooled standard deviation [16] of the first, second, and third follow-up, respectively. The data were analyzed using IBM SPSS Statistics version 27 (IBM Corp., Armonk, NY). The significance level was set at p<0.05.

## Results

All 52 participants completed the study as per the initially planned protocol. The baseline characteristics of the participants in the intervention and control groups are presented in Table 1. It was observed that except for the physical score for WHOQOL-BREF, there was no significant difference between the intervention and control groups at baseline.

**Table 1:** Baseline characteristics of the study participants WHOQOL-BREF: the World Health Organization Quality of Life Brief Version; VAS: Visual Analog Scale; PSQI: the Pittsburgh Sleep Quality Index; BMI: body mass index; SD: standard deviation

Sl.no	Variables	Control (mean ±SD)	Intervention (mean ±SD)	P-value
1	Age, years	20.77 ±1.48	20.77 ±1.42	1.000
2	Height, cm	156.19 ±11.88	158.85 ±5.44	0.995
3	Weight, kg	52.77 ±14.13	52.08 ±8.85	0.303
4	BMI, kg/m^2^	20.92 ±5.08	20.62 ±2.70	0.303
5	Waist circumference, cm	79.65 ±12.15	79.50 ±7.73	0.493
6	Hip circumference, cm	94.50 ±9.89	90.92 ±12.65	0.722
7	WHOQOL-BREF - physical	54.77 ±16.72	42.69 ±11.06	0.043
8	WHOQOL-BREF - psychological	48.42 ±18.71	47.31 ±11.32	0.995
9	WHOQOL-BREF - social	59.58 ±18.38	65.88 ±12.55	0.171
10	WHOQOL-BREF - environmental	62.88 ±18.46	64.35 ±13.52	0.918
11	PSQI	7.69 ±3.38	7.92 ±2.56	0.493
12	VAS	6.69 ±1.59	6.85 ±1.41	0.722
13	Cramps	2.15 ±0.73	2.50 ±0.65	0.493
14	Nausea	0.69 ±0.93	0.88 ±0.99	0.722
15	Dizziness	1.12 ±0.82	1.38 ±0.90	0.995
16	Mood changes	2.27 ±0.87	2.42 ±0.70	1.000
17	Diarrhea	0.92 ±1.44	0.54 ±0.86	0.995
18	Absenteeism	0.85 ±1.01	1.12 ±0.82	0.303

Effects of therapeutic fasting on pain related to DP

The experimental group showed a significant reduction in pain compared to the control group (p<0.001). This effect persisted even after the second (p=0.002) and third (p=0.003) follow-ups, where the experimental group participants reported significantly lower pain intensity compared to the baseline.

Effects of therapeutic fasting on cramps, nausea/vomiting, dizziness, mood, and diarrhea

The experimental group showed a significant reduction in cramps (p=0.001), nausea/vomiting (p=0.02), dizziness (p=0.007), and mood changes (p=0.005) compared to the control group. Even though the experimental group also showed a mean reduction in diarrhea post-intervention compared to the control group, the changes were not statistically significant. The effect was consistent in the experimental group for dizziness (p=0.001, p=0.01) and mood changes (p=0.008, p=0.02) during the second and third follow-ups as well. The experimental group showed a further reduction in cramps compared to the controls at the second follow-up (p=0.001), whereas there was a slight increase in the mean values for cramps in the experimental group during the third follow-up. However, this change was not statistically significant (p=0.06).

During the second (p=0.04) and third (p=0.004) follow-ups, we found a significant increase in diarrhea in the experimental group. Furthermore, we did not observe any significant changes in nausea and vomiting symptoms during the second and third follow-ups.

Effects of therapeutic fasting on QoL

The experimental group's physical (p=0.005) and psychological (p<0.001) QoL improved significantly when compared to the controls. The changes were consistent for psychological QoL during the second (p=0.001) and third (p=0.002) follow-ups as well; however, the physical QoL showed significant improvement only during the second follow-up (p=0.03). The social and environmental QoL domains did not show any significant improvement post-intervention, whereas we noticed a significant reduction in the social QoL of the experimental group during the third follow-up (p=0.02).

Effects of therapeutic fasting on sleep

The experimental group showed a mean reduction in the PSQI scores during the post-intervention assessment and follow-up and a slight increase in the PSQI scores during the third follow-up. However, these changes were not statistically significant.

Effects of therapeutic fasting on absenteeism

The experimental group had significantly lower absenteeism compared to the control group post-intervention (p=0.001). The trend was consistent during the second and third follow-ups, where there was lower absenteeism due to dysmenorrhea in the experimental group compared to the controls. However, this change was not statistically significant. The detailed results are outlined in Tables 2, 3.

**Table 2:** Immediate effects of therapeutic fasting on the study variables compared to the normal daily routine *Analysis of covariance. ^#^Independent samples Mann-Whitney U test WHOQOL-BREF: the World Health Organization Quality of Life Brief Version; VAS: Visual Analog Scale; PSQI: the Pittsburgh Sleep Quality Index; NRS: Numerical Rating Scale; SD: standard deviation

Variables	Baseline (mean ±SD)	Post-intervention (mean ±SD)	Effect size	P-value
VAS pain*				
Control	6.69 ±1.59	6.65 ±1.74	0.02	
Intervention	6.85 ±1.41	4.35 ±2.15	1.38	0.000
WHOQOL-BREF - physical*				
Control	54.77 ±16.72	55.19 ±14.46	0.03	
Intervention	42.69 ±11.06	57.09 ±13.68	1.16	0.005
WHOQOL-BREF - psychological*				
Control	48.42 ±18.71	50.85 ±17.59	0.13	
Intervention	47.31 ±11.32	65.15 ±17.15	1.23	0.000
WHOQOL-BREF - social*				
Control	59.58 ±18.38	60.12 ±18.84	0.03	
Intervention	65.88 ±12.55	70.23 ±16.10	0.30	0.086
WHOQOL-BREF - environmental*				
Control	62.88 ±18.46	64.69 ±14.15	0.11	
Intervention	64.35 ±13.52	66.96 ±13.75	0.19	0.458
PSQI*				
Control	7.69 ±3.80	7.27 ±3.40	0.12	
Intervention	7.92 ±2.56	6.04 ±3.59	0.60	0.157
NRS symptoms - cramps^#^				
Control	2.15 ±0.73	2.19 ±0.63	0.06	
Intervention	2.50 ±0.65	1.50 ±0.81	1.36	0.001
NRS symptoms - nausea/vomiting^#^				
Control	0.69 ±0.93	0.58 ±0.81	0.13	
Intervention	0.88 ±0.99	0.46 ±0.71	0.49	0.025
NRS symptoms - dizziness^#^				
Control	1.12 ±0.82	1.19 ±0.75	0.09	
Intervention	1.38 ±0.90	0.65 ±0.75	0.88	0.007
NRS symptoms - mood changes^#^				
Control	2.27 ±0.87	2.35 ±0.69	0.10	
Intervention	2.42 ±0.70	1.65 ±0.94	0.93	0.005
NRS symptoms - diarrhea^#^				
Control	0.92 ±1.44	0.65 ±0.98	0.22	
Intervention	0.54 ±0.86	0.19 ±0.49	0.50	0.071
Absenteeism^#^				
Control	0.85 ±1.01	1.04 ±1.22	0.17	
Intervention	1.12 ±0.82	0.46 ±0.71	0.86	0.005

**Table 3:** Comparison of changes during the follow-up periods between the fasting group and controls *Analysis of covariance.^ #^Independent samples Mann-Whitney U test WHOQOL-BREF: the World Health Organization Quality of Life Brief Version; VAS: Visual Analog Scale; PSQI: the Pittsburgh Sleep Quality Index; NRS: Numerical Rating Scale; SD: standard deviation

Variables	Intervention (mean ±SD)	Control (mean ±SD)	Effect size	P-value
VAS pain*				
First follow-up	4.35 ±2.15	6.52 ±1.64		
Second follow-up	4.12 ±2.10	5.92 ±1.73	0.94	0.002
Third follow-up	4.31 ±1.80	6.20 ±1.80	1.05	0.003
WHOQOL-BREF - physical*				
First follow-up	62.62 ±13.68	54.16 ±13.74		
Second follow-up	64.27 ±13.72	56.16 ±12.80	0.76	0.034
Third follow-up	63.81 ±16.39	55.80 ±14.46	0.51	0.071
WHOQOL-BREF - psychological*				
First follow-up	65.15 ±17.15	49.64 ±16.82		
Second follow-up	65.42 ±16.39	50.92 ±14.07	0.95	0.001
Third follow-up	66.88 ±18.46	51.32 ±14.51	0.94	0.002
WHOQOL-BREF - social*				
First follow-up	70.23 ±16.10	59.76 ±19.13		
Second follow-up	70.19 ±14.01	63.48 ±14.91	0.46	0.104
Third follow-up	70.92 ±13.43	60.72 ±18.25	0.64	0.027
WHOQOL-BREF - environmental*				
First follow-up	66.96 ±13.75	64.76 ±14.44		
Second follow-up	69.54 ±17.12	63.52 ±14.08	0.38	0.178
Third follow-up	71.50 ±16.88	65.56 ±14.11	0.38	0.180
PSQI*				
First follow-up	6.04 ±3.59	7.08 ±3.33		
Second follow-up	6.19 ±3.42	7.00 ±4.87	0.19	0.500
Third follow-up	6.12 ±3.30	7.40 ±4.10	0.34	0.220
NRS symptoms - cramps^#^				
First follow-up	1.50 ±0.81	2.16 ±0.62		
Second follow-up	1.31 ±0.74	2.00 ±0.65	0.99	0.001
Third follow-up	1.58 ±0.95	2.04 ±0.79	0.53	0.062
NRS symptoms - nausea/vomiting^#^				
First follow-up	0.46 ±0.71	0.56 ±0.82		
Second follow-up	0.31 ±0.55	0.56 ±0.92	0.33	0.473
Third follow-up	0.62 ±0.80	0.56 ±0.82	0.07	0.738
NRS symptoms - dizziness^#^				
First follow-up	0.65 ±0.80	1.16 ±0.75		
Second follow-up	0.42 ±0.64	1.20 ±0.91	0.99	0.001
Third follow-up	0.62 ±0.80	1.20 ±0.87	0.69	0.017
NRS symptoms - mood changes^#^				
First follow-up	1.65 ±0.94	2.32 ±0.69		
Second follow-up	1.62 ±0.98	2.32 ±0.69	0.83	0.008
Third follow-up	1.54 ±0.99	2.16 ±0.80	0.67	0.024
NRS symptoms - diarrhea^#^				
First follow-up	0.19 ±0.49	0.64 ±1.00		
Second follow-up	0.23 ±0.51	0.68 ±0.95	0.59	0.047
Third follow-up	0.15 ±0.37	0.76 ±0.93	0.86	0.004
Absenteeism^#^				
First follow-up	0.46 ±0.71	0.92 ±1.08		
Second follow-up	0.50 ±0.71	0.64 ±1.04	0.16	0.913
Third follow-up	0.54 ±0.76	0.60 ±0.82	0.08	0.829

Symptoms associated with fasting

Apart from four participants, all the other participants reported that they had experienced at least one adverse symptom during fasting. Fatigue (n=17) was the most commonly reported symptom during fasting, followed by headache (n=13), body ache (n=11), constipation (n=11), giddiness (n=8), fever (n=7), sleep disturbance (n=7), vomiting (n=4), and hyperacidity (n=2). Of note, 19 participants reported having experienced more than one symptom (one symptom: n=3, two symptoms: n=5, three symptoms: n=4, four symptoms: n=3, five symptoms: n=2, six symptoms: n=2, and seven symptoms: n=3). None of the participants discontinued the fasting or required emergency medical care due to the symptoms associated with therapeutic fasting.

## Discussion

In the present study, we evaluated the long-term impact of prolonged therapeutic fasting on young female adults with PD. The main findings were as follows: (i) therapeutic fasting offers long-term pain relief in dysmenorrhea; (ii) therapeutic fasting reduces symptoms like cramps, dizziness, and mood changes associated with dysmenorrhea; and (iii) therapeutic fasting improves the physical and psychological QoL and decreases absenteeism in young females with dysmenorrhea.

Severe pelvic pain is reported to be a common symptom among young female adults with dysmenorrhea [17]. The participants in the experimental arm of the present study showed a consistent reduction in pain after their exposure to fasting throughout the study period. This indicates the role of fasting in providing a long-term analgesic effect for up to three months. The analgesic effect of fasting in dysmenorrhea may be attributed to the heightened parasympathetic activity following the fasting period and its impact on stress hormones like cortisol, which is reported to decrease post-fasting [18]. Furthermore, severe dysmenorrhea is associated with dietary patterns, and a low-fat vegetarian diet has been shown to reduce the intensity and duration of dysmenorrhea [19,20].

In our study, the participants in the experimental arm reported a reduction in their symptoms associated with dysmenorrhea, such as cramps, nausea/vomiting, dizziness, mood changes, and diarrhea. The decrease in cramps might be attributed to the way therapeutic fasting affects the levels of the uterine prostaglandins (PGF2 and PGE2) that cause cramps and pain in females with dysmenorrhea [1,8]. The effect of fasting on cramps lasted up to two months, indicating that therapeutic fasting can be used as a long-term treatment strategy for dysmenorrhea.

PD is associated with poor QoL in women and has been shown to significantly affect their day-to-day activities, mood, and contentment [21,22]. Our data suggest that therapeutic fasting can significantly improve the physical and psychological QoL. While the impact of fasting on physiological QoL lasted for two consecutive menstrual cycles, psychological QoL consistently improved throughout the trial period. This indicates that periodic fasting once every two months may be beneficial in preserving both physical and psychological QoL in patients with dysmenorrhea.

The improvement in QoL correlates with the improvement in the mood of our study participants, who reported an improvement in their mood after fasting. Our findings are in agreement with a previous study that reported that fasting improves mood and QoL [23]. However, this is the first study to report the impact of fasting on QoL in young adults with PD. Fasting induces emotional conduciveness due to its effect on neurotrophic factors and neurotransmitters, which regulate pain sensation [24], as well as the increase in plasma endorphin levels [25].

Dysmenorrhea has been reported to be one of the significant reasons for absenteeism in schools and colleges among young female adults [26]. Frequent absenteeism is also linked to academic underachievement among young adults. Our findings point to a significant decrease in absenteeism during the first month following the fasting, as well as improvements in the second and third follow-up months. This study postulates that fasting could be a potential public health tool for reducing absenteeism associated with dysmenorrhea.

The majority of the study participants (92%) reported having experienced symptoms like fatigue, headache, fever, giddiness, constipation/diarrhea, and sleep disturbances. However, these symptoms were self-limiting, and all the participants were able to successfully complete the 10-day fasting program without any serious adverse events. Furthermore, these symptoms are deemed to be expected reactions to fasting as per the international expert consensus on fasting therapy [10]. The present data provide insights into the expected reactions to therapeutic fasting, which may improve preparedness while designing fasting programs. Nevertheless, therapeutic fasting was found to be safe among our study participants.

To our knowledge, this is the first clinical trial to report the impact of therapeutic fasting on PD. All the participants in the present study adhered to the fasting protocol for the entire study period of 10 days. Our study establishes the long-term impact of therapeutic fasting on both physical and psychological symptoms associated with PD. The impact of fasting on prostaglandins, cortisol, brain-derived neurotrophic factor (BDNF), and the autonomic nervous system could explain the beneficial effects of therapeutic fasting on PD. Future research may look into these aspects to better understand the mechanisms underlying therapeutic fasting's clinical effectiveness in the treatment of dysmenorrhea.

The present study is limited in terms of its assessment of the role of specific food items consumed while fasting among dysmenorrhea patients, as the primary intention of the investigators was to provide a low-calorie, low-fat vegetarian diet. This may be considered a limitation. Future studies may investigate the role of specific food choices during fasting in PD patients.

## Conclusions

This study endorses the practice of therapeutic fasting as a potential non-pharmacological therapeutic option for young female adults with PD. Based on our findings, therapeutic fasting can progressively reduce pain, cramps, and mood swings and improve QoL in young adults with PD. However, the mechanisms by which therapeutic fasting modulates clinical outcomes have not been explored in the present study. Hence, further follow-up studies with larger sample sizes and advanced clinical markers are warranted to build on the present findings.

## Appendices

**Table 4:** Summary of follow-up instructions

Post-fasting follow-up dietary routine	
Dos	Consume a plant-based vegetarian diet. Maintain a balance between the consumption of raw and cooked foods. Increase the quantity of fruits, salads, and sprouts in their diet. Drink plenty of fluids, at least 3–4 liters (water, vegetable and fruit juices, etc.). Eat homemade recipes and decrease your consumption of outside-prepared food. Eat at proper times with a feeding interval of 8–12 hours
Don'ts	Decrease consumption of outside-prepared food. Avoid deep-fried and overcooked foods. Avoid packed, tinned, refined, and processed foods. Avoid white sugar, candies, cold drinks, and soft drinks. Avoid binge-eating. Avoid eating during emotional outbursts
